# Implementation challenges of free maternity services policy in Kenya: the health workers’ perspective

**DOI:** 10.11604/pamj.2015.22.375.6708

**Published:** 2015-12-16

**Authors:** Emmanuel Wekesa Wamalwa

**Affiliations:** 1 Department of Health Systems Management, Kenya Methodist University

**Keywords:** Free maternity services policy, implementation challenges, recommendations

## Abstract

**Introduction:**

Kenya implemented the policy of free maternity services to reduce maternal mortality and morbidity. For successful implementation of this policy, there is need to monitor the implementation progress, identify the challenges and mitigate them and determine better strategies for implementation based on emerging implementation issues. This study was carried out to determine the challenges facing policy implementation and strategies for better implementation.

**Methods:**

This was a cross-sectional descriptive study carried at the Rift Valley Provincial General Hospital (RVPGH) and Bondeni maternity. All the staff who work at Bondeni Maternity (including nursing officer in-charge) were included in the study. All the staff who work at the Maternity Unit of the RVPGH were included in the study, as well as the Medical Superintendent of the facility. A total of 110 respondents were sampled. A questionnaire and interview guide were used to collect data. Data was analyzed using SPSS software.

**Results:**

A response rate of 91% (n=100) was achieved. Major implementation challenges include inadequate supplies (86%), inadequate funding (38%), staff shortage (92%), lack of motivation among health workers (62%), overwhelming workload (89%) and abuse of services by clients (32%). Strategies for better implementation include employment of more staff, motivation of health workers, improvement in policy financing, training of health workers and provision of adequate supplies.

**Conclusion:**

Implementation of free maternity services policy in Kenya is facing challenges but there exists strategies, which, if implemented, will help address these challenges.

## Introduction

Kenya is one of the countries considered to be having a high maternal mortality rate. According to the Kenya Demographic and Health Survey (KDHS) 2008-2009 report, the national maternal mortality ratio is 488 per 100,000 live births. This is partly contributed by limited access to maternal health services. Evidence on user fees and other out-of-pocket payments in Kenya revealed that health care charges were a significant barrier to access of maternal health services especially among the poorest populations [1]. According to the KDHS 2008-2009, about 43 percent of births in Kenya take place in a health facility while 53 percent of deliveries take place at home. Ensuring all women give birth with a skilled birth attendant and access to emergency obstetric care is accepted as the most crucial intervention for reducing maternal and newborn deaths [2]. Unfortunately, studies have shown that this strategy is largely hampered by user fees in low and middle income countries. It has further been acknowledged that user fees in public facilities contribute to catastrophic expenses [3], reduce health services utilization [4] and are a major impediment to universal coverage [5]. Findings of studies on exemption policy in Ghana confirmed that introduction of free delivery care policy is associated with an increase in facility deliveries [6]. Many other low and middle-income countries have initiated user fees exemption policies as a method of increasing financial access to health care. The Government of Kenya rolled out free maternity services program on 1st June, 2013 through a presidential declaration to encourage women to give birth at health facilities under skilled personnel. This was in keeping with the resolutions of the African Union favoring point-of-service user fees exemptions for pregnant women and children under the age of five years [7]. The policy aims at reducing maternal complications as well as maternal mortalities in Kenya. The program is meant to eliminate all the charges for intra-partum care in public health facilities. The government allocated KShs. 4 billion (USD 44.4 million) in the 2013/2014 budget for implementation of the program. Under this program, the health centers and dispensaries should be reimbursed Kshs. 2,500 (USD 27.7) for every delivery, through the Hospital Sector Services Fund. The hospitals should be reimbursed KShs. 5,000 (USD 55.6) for every delivery conducted in the facility, through the Hospital Management Service Fund. In addition, the government set aside KShs. 3.6 billion (USD 40 million) to hire 7,500 additional health workers to cope with the expected increase in workload. To ensure successful implementation of such a program, there is need for monitoring and evaluation of implementation progress. This ensures that implementation is on course and any arising challenges are mitigated in time. There are no documented studies done to monitor progress of implementation. This study was carried out to evaluate implementation, focusing on challenges and possible remedies for these challenges. The objective of the study was to determine challenges facing implementation of free maternity services policy and determine strategies for improving implementation, based on health workers’ perspective.

## Methods

A descriptive cross-sectional study design was employed in this research. The study focused on two health facilities in Nakuru County (The Rift Valley Provincial General Hospital and Bondeni Maternity). The target population was the health workers who are involved in implementation of the policy. They included doctors, clinical officers and nurses. A census was used to select the respondents from the sampled health facilities. All the health workers who are stationed at the maternity unit of the Rift Valley Provincial General Hospital were included in the study. All the health workers in Bondeni Maternity were included in the study. The Medical Superintendent in charge of the Rift Valley Provincial General Hospital as well as the Nursing Officer in charge of Bondeni Maternity were also included in the study as key informants. The sample size comprised of 110 respondents obtained from the two health facilities Table 1. Purposive sampling was used to sample the facilities for the study. The Rift Valley Provincial General Hospital was purposely sampled because it is the largest and main referral hospital in Nakuru County, offering comprehensive obstetric care. Bondeni Maternity was purposely sampled because it is one of the largest health centers in the county recording the highest number of deliveries compared to other health centers. It was necessary to include in the study both a hospital and a health center because implementation of the policy is done at both levels but differently. This implies that the hospitals and health centers face different challenges in the implementation of the policy hence the need to include both in the study. Only public health facilities were included in the study because the policy is meant to be implemented in public health facilities. The questionnaire was used as the main research instrument in the study. The questionnaire was administered to all respondents except the key informants who are the personnel in-charge of the sampled health facilities. An interview guide was used to obtain information from key informants. Qualitative methods were used to complement the quantitative data. The data was collected from 24th April, 2014 to 23rd May, 2014. Analysis of the data was done using SPSS software and Microsoft Office Excel. The degree of association between responses from the two facilities was tested at 95% confidence interval.

**Table 1:** Designations of the study sample from the Rift Valley Provincial General Hospital and Bondeni Maternity

Health Workers	Rift Valley Provincial General Hospital Bondeni Maternity			
	Total (%)	Sampled (%)	Total (%)	Sampled (%)
Doctors	12 (12.8)	12 (12.8)	0 (0)	0 (0)
Clinical Officers	19 (20.1)	19 (20.1)	0 (0)	0 (0)
Nurses	62 (66)	62 (66)	15 (93.8)	15 (93.8)
Personnel In-charge of facility	1 (1.1)	1 (1.1)	1 (6.2)	1 (6.2)
**TOTAL**	**94 (100)**	**94 (100)**	**16 (100)**	**16 (100)**

Ethical Considerations: ethical Clearance to carry out the research was obtained from Kenya Methodist University's Board of Scientific and Ethics Review Committee. Authorization to conduct the study at the Rift Valley Provincial General Hospital was given by the Medical Superintendent following perusal and approval by the research committee of the facility. A written authorization to conduct the study at Bondeni Maternity was given by the Nakuru County Director of Health. Confidentiality was observed during the study. The identity of the respondents was withheld. The codes were used to identify the respondents.

## Results

**Characteristics of respondents**: a total 100 (91%) respondents participated in the study out of the sampled 110, giving an overall response rate of 91%. The respondents included doctors, clinical officers and nurses from the Rift Valley Provincial Hospital and Bondeni Maternity. Majority of the respondents were nurses, accounting for 72% (n=72) of all the respondents. This is because majority of the maternal health services in health facilities are offered by nurses. In addition, majority of the respondents, 85% (n=85) were female. This is because majority of the respondents were nurses who were female. Regarding the experience of the respondents in the provision of maternal health services, majority, 48% (n=48) had the experience of more than five years. Others had an experience of 1-5 years (29%) and less than one year (23%).

**Challenges Facing Implementation of Free Maternity Services Policy**: the study sought to determine the challenges facing implementation of free maternity services policy, based on the health workers’ perspective. This was important because health workers being implementers of the policy are better placed to understand these challenges. All the respondents (n=100) indicated that they were facing implementation challenges. The respondents were further asked to specify the challenges. Majority of the respondents indicated that there was staff shortage (92%), overwhelming workload (89%) and inadequate supplies (86%). Other challenges included abuse of the free services (32%), uncooperative clients (26%) financial challenges (38%) and lack of managerial support (18%). The key informants (personnel in charge of the facilities) highlighted inadequate financing as a major challenge. The medical superintendent of the Rift Valley Provincial General Hospital indicated that the facility was irregularly reimbursed 50-74% of the expected funds. The Nursing Officer in-charge of Bondeni Maternity indicated that the facility had never received any reimbursements since the program was rolled out. A comparison of responses regarding challenges facing implementation of free maternity services policy in the two facilities was made. Most of the challenges (lack of supplies, inadequate skills among health workers, staff shortage, overwhelming workload, and rude clients) experienced by the health care workers in the two facilities were similar statistically. However, financial challenges, abuse of free services and lack of managerial support differed significantly (p=0.029, 0.03, 0.001 respectively). Majority (64.3%) of staff at Bondeni Maternity reported that the facility experienced financial challenges as compared to 33.7% of the staff at Nakuru PGH who reported the same challenge. Similarly Abuse of free services by the public was reported in Bondeni Maternity by 57.1% (n=8) as compared to only 27.9% (n=24) who reported the same in Nakuru PGH Table 2. Motivation of health workers to implement the policy is an important success factor. The study found that majority of the respondents, 62% (n=72) were not motivated to implement the policy. Lack of motivation was attributed to the implementation challenges being faced as well as increased workload arising from increased utilization of maternal health services in public health facilities. All respondents (n=100) reported increased workload since introduction of free maternity services policy. However, there was no difference in the level of motivation among health workers in the two facilities (p=0.825). Lack of support from the government was also found to be a major implementation challenge. Respondents were asked whether the government was doing enough to support implementation of the policy. Majority (90%) indicated that the government was not doing enough to support implementation while 10% of the respondents reported that the government was doing enough to support the policy.

**Table 2:** Comparison of implementation challenges between the two facilities

		Facility		P-values
		NakuruPGH (%)	Bondeni Maternity (%)	
Financial challenges	Yes	29 (33.7)	9 (64.3)	0.029
	No	57 (66.3)	5 (35.7)	
Supplies challenges	Yes	72 (83.7)	14 (100)	0.208
	No	14 (16.3)	0 (0.0)	
Health workers inadequate skills	Yes	14 (16.3)	3 (21.4)	0.702
	No	72 (83.7)	11 (78.6)	
Staff shortage challenges	Yes	78 (90.7)	14 (100)	0.596
	No	8 (9.3)	0 (0.0)	
Overwhelming work	Yes	77 (89.5)	12 (85.7)	0.65
	No	9 (10.5)	2 (14.3)	
Rude client	Yes	24 (27.9)	2 (14.3)	0.346
	No	62 (72.1)	12 (85.7)	
Abuse of free services	Yes	24 (27.9)	8 (57.1)	0.03
	No	62 (72.1)	6 (42.9)	
Lack of managerial support	Yes	9 (10.5)	9 (64.3)	0.001
	No	77 (89.5)	5 (35.7)	

**Health workers’ recommendation for better implementation**: The respondents recommended to the government the various strategies that can be employed to enhance motivation of health workers. The data collected showed that improvement of remuneration was among the key strategies recommended by health workers (49%) to enhance motivation of implementers. Seventeen percent (n=17) of the respondents indicated that improving working conditions was necessary to motivate health workers. This included reduction in number of working hours, improving working environment and provision of incentives. Other recommendations included improvement of supplies, increasing number of staff involved in implementation, training opportunities for staff to enhance skills and improved supervision from management. The health workers recommended various strategies that the government may initiate to improve implementation of the program. The most advocated recommendation was an increase in the number of staff in health facilities by 74% of the respondents, improved supplies by 57% of the respondents, provision of training opportunities (23%) and improving infrastructure (20%).

## Discussion

Staff shortage was reported as a major implementation challenge, according to 92% (n=92) of the respondents. Key informant interview revealed that the government never implemented its plan of employing more staff to cope with increased utilization of maternal health services. The facilities had not received any additional staff to cope with the increasing workload. This partly accounted for the challenge of overwhelming workload. Financial challenges were cited as the main reason for lack of supplies. This was further emphasized by personnel in-charge of the two health facilities. The personnel in-charge of Rift Valley Provincial General Hospital reported that the facility received only 50-74% of the reimbursements and on irregular basis. The Nursing Officer In-charge of Bondeni Maternity reported that the facility has never received any reimbursements from the government since the introduction of the policy. The officer reported that this challenge has greatly affected the quality of care. Underfunding was also seen as a challenge in other countries that have implemented such a policy. In Ghana for instance the policy was underfunded by 34% in 2004 and 73% in 2005 [8]. Due to underfunding, governments sometimes had to reintroduce user fees after they had been abolished. In Senegal, health centres increased the fees for certain services that could still be charged in order to compensate for lost income from those services that had become free [9]. The inadequate funding flows create friction between communities and health staff and between facility managers and higher levels of the health system and this can potentially cause program failure. Underfunding can cause implementation failure of the policy leading to out-of-pocket payments for ‘free’ services. For instance in Tanzania 73.3% of women with facility delivery reported having made out-of-pocket payments for delivery-related costs after abolition of user fees [10].

Shortage of supplies was cited as a major challenge by majority of the respondents (86%). The shortage of supplies was occasioned by underfunding of the policy. The findings were consistent with the findings of investigators in other countries which have implemented such a policy, including Madagascar and South Africa [11]. The shortage of supplies can adversely undermine the implementation of the policy. In fact, some authors reported that health workers perceived the drastic decline in service utilization after seven months of exemption to be a consequence of the shortages in additional drugs and other supplies [12]. Majority of the respondents (62%) are not motivated to implement the free maternity services policy in their health facilities while 38% are motivated. Lack of motivation was therefore found to be one of the main challenges facing implementation of the policy. Evidence from other countries suggests that failure to boost workforce during implementation of such a policy can lead to reduced morale and motivation. In Uganda for instance there was health workforce crisis following introduction of user fee exemption program, characterized by a decline in health workers’ morale, negative attitude towards the program, heavier workload for health workers and feeling of inadequacy among the health personnel [13]. A study done in Afghanistan found that the morale of the health workers reduced due to loss of discretionary revenue [14]. Similar findings were noted in Niger [15]. Lack of motivation to implement the policy should therefore be seen as a major challenge to implementation since motivation has shown to be a major factor for successful implementation. The respondents also recommended to the government the various strategies that can be employed to enhance motivation of health workers. Majority of respondents (49%) recommended motivation of health workers through reduction in number of hours, improving work environment and provision of incentives. Other recommendations included improvement of supplies, increasing number of staff involved in implementation, training opportunities for staff to enhance skills and improved supervision from management. The findings were partly in keeping with the findings from other investigators in other countries. In Ghana for instance, a study done by [8] reported that health workers’ motivation to implement such policies included opportunity to serve the community (66%), social status attached to the profession (8.3%), opportunities for training (4.3%) and allowances (3.8%).

In another study conducted in Ghana, health workers gave the following recommendations to improve implementation of free delivery services policy: regular release of reimbursement funds, improved working conditions for staff, provision of more logistics and supplies, and training and recruitment of more staff [8]. The study recommends that the Ministry of Health, in cooperation with the Nakuru County Government should employ additional staff to cope with the shortage of staff and increased workload occasioned by increased utilization of maternal health services in public health facilities. In this regard, an evaluation of human resource requirements needs to be done to determine the staffing needs of public facilities involved in the implementation of the policy. The evaluation should be followed by employment of more staff to eradicate the current shortage. This intervention will also help improve the quality of service. The study recommends that the Ministry of Health should improve on its financial systems so as to promptly disburse needed funds which have been noted as an implementation challenge for this policy. In this regard, a review of the disbursement mechanisms and procedures is also recommended. An evaluation of adequacy of funding, by the Ministry of Health, is also recommended to determine whether current reimbursements are adequate for successful policy implementation. Motivation of health workers to implement the policy is also recommended in this study. The Ministry of Health, in conjunction with County Government should come up with initiatives to motivate those health workers who implement the programme. The initiatives may include allowances, training opportunities in maternal health services and supportive supervision. Further research is recommended to evaluate the actual requirements for implementation of free maternity services policy. The study should focus on infrastructure, human resources, financial and supplies requirements. The findings of the study will help effectively mitigate the challenges facing implementation. Further research is recommended to be conducted on the policy, after at least five years of implementation to evaluate the success and impact of the policy. This was not done in this study because it was too early to do so.

## Conclusion

Regarding implementation challenges, this study concludes that the major challenges include lack of motivation and inadequate funding which is partly caused by partial reimbursements from the government. Underfunding was a major cause of shortage of supplies which was a major implementation challenge. Staff shortages and overwhelming workload are major implementation challenges that result from government's failure to boost the human resource capacity to cope with increased utilization of maternal health services. Regarding strategies for improving implementation, the study concludes that effective implementation strategies include: boosting the human resource capacity through employment of additional health workers to cope with increased workload; motivation of health workers by improving their remuneration and working conditions; improving supplies through increased funding and adequate reimbursements; improving infrastructure to increase the capacity of health facilities to cope with increased number of patients; and supportive supervision of health workers.
